# Trends in mesenchymal stem cell-derived extracellular vesicles clinical trials 2014–2024: is efficacy optimal in a narrow dose range?

**DOI:** 10.3389/fmed.2025.1625787

**Published:** 2025-09-18

**Authors:** Yusong Wang, Junchi Zhu, Qimin Ma, Wei Zhou, Linshan Yang, Shuyue Sheng, Feng Zhu, Zhaofan Xia

**Affiliations:** ^1^Department of Critical Care Medicine, Shanghai East Hospital, School of Medicine, Tongji University, Shanghai, China; ^2^Shanghai Jiao Tong University School of Medicine, Shanghai, China; ^3^Department of Burn Surgery, The First Affiliated Hospital of Naval Medical University, Shanghai, China

**Keywords:** mesenchymal stem cells, extracellular vesicles, exosomes, nebulization, lung diseases, COVID-19, clinical trials

## Abstract

**Background:**

Mesenchymal stem cell-derived extracellular vesicles (MSC-EVs) are emerging as promising cell-free therapeutic agents due to their immunomodulatory and regenerative properties. However, the lack of standardized protocols and dose optimization strategies has limited their clinical translation. While procedures for the isolation, expansion, and therapeutic use of mesenchymal stem cells (MSCs) have been standardized, there remains a lack of standardized protocols for the isolation and purification of EVs and exosomes (Exos).

**Methods:**

This review Comprehensive statistical summary global clinical trials involving MSC-EVs and Exos registered between 2014 and 2024, with a particular focus on dose-effect relationships and administration routes. Data were collected from ClinicalTrials.gov, the Chinese Clinical Trial Registry, and the Cochrane Register of Studies. A total of 66 eligible trials were included after screening.

**Results:**

Intravenous infusion and aerosolized inhalation were identified as the predominant administration methods, especially in trials targeting respiratory diseases. Notably, dose-effect results revealed that nebulization therapy achieved therapeutic effects at doses around 108 particles, significantly lower than those required for intravenous routes. This suggests a relatively narrow and route-dependent effective dose window. However, large variations in EVs characterization, dose units, and outcome measures were observed across trials, underscoring the lack of harmonized reporting standards.

**Conclusion:**

This review highlights dose-response as a critical but underappreciated gap in current MSC-EVs clinical research. The findings emphasize the urgent need for standardized dosing frameworks, potency assays, and harmonized clinical protocols to advance the safe and effective translation of MSC-EVs therapies. The analysis underscores the need for standardized protocols, global collaboration, and a deeper understanding of the biological mechanisms underlying MSC-EVs and Exos therapies to advance clinical applications and ensure safety and efficacy.

## 1 Introduction

Mesenchymal stem cells (MSCs) can be isolated from a variety of tissues, including bone marrow, adipose tissue, umbilical cord, umbilical cord blood, placenta, Wharton’s jelly, amniotic fluid, synovium, saphenous vein, dental pulp, periodontal ligament, cervical tissue, skeletal muscle tissue, lung tissue, liver tissue, and dermal tissue (1, 2). MSCs hold significant therapeutic potential for treating various diseases due to their: (1) differentiation potential-the ability to differentiate into various cell lineages; (2) paracrine effect-secretion of soluble factors essential for cell survival and proliferation; (3) immune regulation-interaction with a variety of immune cells to regulate immune response; and (4) migration and homing-responding to specific signal molecules from damaged tissues to reach and repair them. Extensive preclinical and clinical research has confirmed the development of MSCs therapy for various diseases (1, 3, 4). Recent studies have shown that MSCs are largely influenced by biological factors such as proteins, mRNA, and microRNA rich in extracellular vesicles (EVs), particularly exosomes (Exos) with diameters of 30–150 nm (5–8). These EVs offer advantages such as low immunogenicity, stability, comparable efficacy, and no risk of tumorigenesis or thrombosis (9).

According to the minimum standards proposed by the International Society for Cellular Therapy (ISCT), current procedures for MSCs isolation, expansion, and therapeutic use have been standardized (10, 11). However, standardized protocols for the isolation and purification of EVs and Exos are lacking. Research indicates that the biological functions and characteristics of MSC-EVs and MSC-Exos vary significantly in size, composition, and function depending on their tissue source (12). This inherent variation influences the therapeutic efficacy of the EVs and Exos in different diseases. The most commonly used MSCs sources in clinical trials are bone marrow, adipose tissue, and umbilical cord. Increasingly, clinical studies are evaluating the therapeutic effects of MSC-Exos from different sources across various diseases (3). However, their rapid clearance from the body may limit their long-term therapeutic effects. Furthermore, factors such as the use of different or undisclosed MSCs tissue sources and passage times, isolation and purification protocols, dosage units in clinical trials, and routes of administration contribute to variability. Research registrations often lack comprehensive information about MSC-EVs and MSC-Exos to protect intellectual property, complicating comparisons between clinical trial studies (5). Consequently, there is a need for systematic evaluation of MSC-EVs and MSC-Exos in clinical trials to assess their safety and efficacy, and the lack of standardized application guidelines may hinder their clinical translation.

Based on this context, the first part of this study employed the Cochrane Register of Studies, ClinicalTrials.gov, and the Chinese Clinical Trial Registry to conduct a detailed review of global clinical trials involving MSC-derived EVs and Exos. This review generated the latest dataset of clinical trials and reported results, analyzing administration routes, dose ranges, and outcome reporting-an unprecedented endeavor. The visual data analysis covered aspects such as time, country and region, type, design, phase, indication, MSCs source, and dose of existing clinical trial studies. The study also analyzed dose-effect relationships and outcome reporting separately. By focusing on dose ranges and result reporting, the study aims to identify the dose-effect relationship under different administration methods in lung diseases, which may reduce the long-term cost of clinical trials, increase the reporting of results (including rarely published negative results), reduce unnecessary clinical trial duplications, and avoid potential ineffective doses.

MSC-EVs can be derived from various tissue sources, including bone marrow, adipose tissue, umbilical cord, placenta, and others. Following cell culture, extracellular vesicles are isolated from conditioned media using techniques such as differential centrifugation, ultracentrifugation. Characterization is performed using nanoparticle tracking analysis (NTA), flow cytometry, and electron microscopy to confirm size, concentration, and surface markers. Therapeutically, MSC-EVs have shown promising applications in diverse organ systems, including the brain (neuroprotection), lungs (inflammatory injury and ARDS), liver (fibrosis and regeneration), pancreas (diabetes and pancreatitis), joints (arthritis and cartilage repair), intestines (inflammatory bowel disease), reproductive system (endometrial repair), skin (wound healing), and eyes (corneal regeneration), highlighting their broad regenerative and immunomodulatory potential.

## 2 Materials and methods

### 2.1 Retrieving clinical trial databases

Data were collected from the institutional databases of the Cochrane Register of Studies, ClinicalTrials.gov, and the Chinese Clinical Trial Registry using the search terms “mesenchymal stem cells (MSCs),” “extracellular vesicles (EVs),” and “exosomes (Exos).” The specific search query employed was (“Mesenchymal Stem Cells” OR “MSCs” OR “Mesenchymal Stem Cell”) AND (“Extracellular Vesicles” OR “EVs”) AND (“Exosomes” OR “Exos”). Clinical trials registered from the inception of these databases to 17 February 2024, were included, without restrictions on study start time or research sponsorship.

The data were downloaded into CSV files and included the following items: registration number, trial title, recruitment status, sponsor, clinical stage, country of origin, and registration date. Additional information, such as disease targets, cell sources, routes of administration, and doses, was manually extracted from the trial registration records when it could not be directly matched and downloaded from the databases. Due to incomplete data in many categories, a different number of trials may be included in each parameter analyzed. Duplicate clinical trials were identified and eliminated based on registration numbers before entering the subsequent analysis process (Table 1).

**TABLE 1:** Database search results (search date: 2024-2-17).

Database	Search query	Search results
Cochrane Register of Studies	(“Mesenchymal Stem Cells” OR “MSCs” OR “Mesenchymal Stem Cell”) AND (“Extracellular Vesicles” OR “EVs”) AND (“Exosomes” OR “Exos”)	110
ClinicalTrials.gov	(“Mesenchymal Stem Cells” OR “MSCs” OR “Mesenchymal Stem Cell”) AND (“Extracellular Vesicles” OR “EVs”) AND (“Exosomes” OR “Exos”)	61
Chinese Clinical Trial Registry	(“Mesenchymal Stem Cells” OR “MSCs” OR “Mesenchymal Stem Cell”) AND (“Extracellular Vesicles” OR “EVs”) AND (“Exosomes” OR “Exos”)	16

### 2.2 Inclusion and exclusion criteria for clinical trials

The inclusion criteria for clinical trials in the search results are:

1. Clinical studies that have completed full registration.
2. Studies using MSC-derived EVs or Exos as the main intervention method.
3. Projects that meet the international ethical and scientific quality standards for clinical trials (Good Clinical Practice, GCP).

The exclusion criteria for clinical trials are:

1. Repeated clinical trials.
2. Projects with withdrawn registration status.
3. Projects with supplementary terms registration type.
4. Projects that do not use MSC-derived EVs or Exos as the main ingredients of the intervention drug.
5. Projects that have not undergone full registration or ethical review to obtain clinical trial qualifications.

**FIGURE 1:**
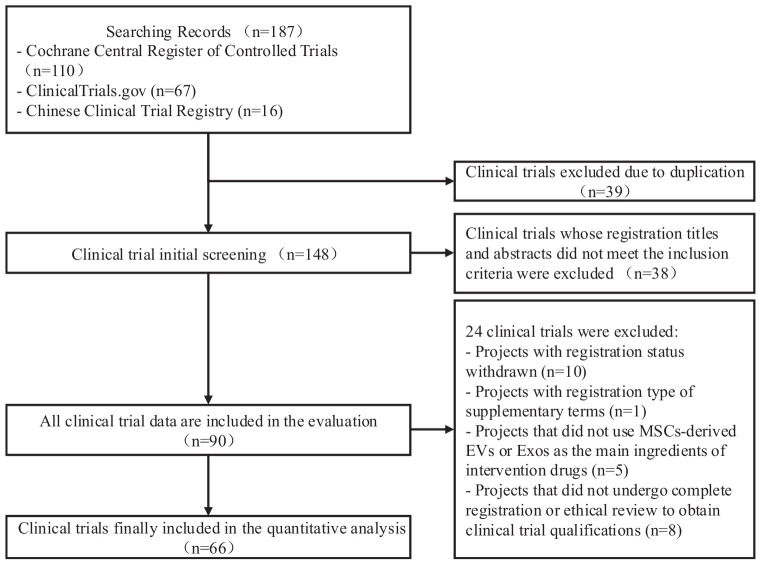
Clinical trial screening flow chart.

The screening process is illustrated in Figure 1.

### 2.3 Screening and confirmation of clinical trials

The clinical trial research data collection was conducted by two trained researchers. After retrieving all clinical trials, two independent researchers screened the included trials based on the duplicate data and the inclusion and exclusion criteria. Controversial clinical trial research was referred to a third trained researcher for resolution. This process ensured the final determination of the clinical trial projects included in this systematic visualization analysis.

### 2.4 Information extraction

Three independent researchers evaluated all clinical trial titles and research statuses. Controversial parts were resolved through communication and discussion among the three researchers. The extraction of relevant information from the final included clinical trial studies encompassed: clinical trial registration number, study start year, country and unit, study type, study phase, study design, route of administration, disease and injury type, source of exosomes or extracellular vesicles, drug dosage description or treatment regimen, publication of study results and Study URL. The latest dataset is generated in the Supplementary Appendix Tables 1, 2.

### 2.5 Comprehensive evaluation of visual analysis results and dose-effect reporting

Power BI 2.1 software was used to generate datasets based on the research detailed in Supplementary Appendix Tables 1, 2. These visualized various aspects of clinical trial research, including time, country and region, type, design, stage, indication, and source of MSCs. The research stage classifications were based on the descriptions of clinical trial stages by the China Center for Food and Drug International Exchange (CCFDIE) and the National Medical Products Administration (NMPA), categorizing exploratory trials/preliminary experiments as Phase I/Phase II clinical research.

Clinical trials with reported results were identified, and these results were not limited to publication in registration databases, journal literature, journal news, or open access sources. Clinical trials were further categorized according to the route of administration and the type of injury or disease. For clinical trial studies that published application doses and results, the main components, doses, frequency of administration, and effects of the intervention drugs were statistically analyzed.

Due to differences in the unit modes of MSC-EVs or MSC-Exos dose calculations, a unified standard was adopted wherever possible. Tables 1, 2 were summarized based on tolerance, safety, adverse events, and dose-effect trends to provide a comprehensive evaluation of the dose-effect relationship.

**TABLE 2:** Summary of dose-effect relationship in representative MSC-EV clinical trials.

Registration No.	Disease/indication	Population (*n*)	Source	Characterization reported	Administration route	Dose (unit)	Regimen	Outcomes summary	AEs reported
NCT04493242	Moderate-severe ARDS (COVID-19)	50	BM-MSC (ExoFlo^®^)	Not detailed	IV infusion	10 mL/15 mL (∼10^10^ EVs/mL)	Days 1 and 4 (2 doses total)	Mortality (RR = 0.385, *p* = 0.034); ↑ Ventilation-free days (*p* = 0.045); subgroup benefits observed	None
NCT04313647	Healthy volunteers	24	hAD-MSC	Not reported	Nebulization	2–16 × 10^8^ particles	Single dose	All tolerated well; transient sinus bradycardia (2/24, not dose-dependent)	Mild (bradycardia)
NCT04276987	COVID-19 pneumonia	7	hAD-MSC	Not reported	Nebulization	2 × 10^8^ daily × 5 days	5 consecutive daily doses	Good tolerance; CT improved by Day 7; no dose-relevant toxicity observed	None
NCT03857841	Not specified	Not available	Not specified	Not reported	IV infusion	20/60/200 Pmol/kg (dose-escalation)	Single dose	Necrotizing colitis in 1/2 at 20 Pmol/kg → trial discontinued	Serious AEs
NCT04491240	COVID-19 pneumonia	Not available	hUC-MSC	Not reported	Nebulization	0.5–2 × 10^10^ particles	BID for 10 days	CRP significantly decreased; no adverse events	None
IRCT20200217046526N2	COVID-19 pneumonia	43	hUC-MSC	Not reported	IV+ nebulization	100 × 10^6^ cells ± MSC-EVs (dose not specified)	Single or dual route combo	MSC-EVs group: 0% mortality vs. 33% in control; ↓ IL-6, TNF-α, IFN-γ, CRP	None

AEs, adverse events; CRP, C-reactive protein; RR, relative risk; EV, extracellular vesicles; IV, intravenous; BID, twice daily.

## 3 Results

### 3.1 Clinical trial search results

Data Collection: The trial item data were downloaded into CSV files and included registration number, trial title, recruitment status, sponsor, clinical stage, country of origin, and registration date. Additional information about the disease, source of MSCs, route of administration, and dose was manually extracted from the trial registration data, as it could not be directly matched and downloaded from the databases. In many cases, complete data for all these categories were not found, but data were collected whenever possible, resulting in different numbers of trials included in each parameter analyzed.

**FIGURE 2:**
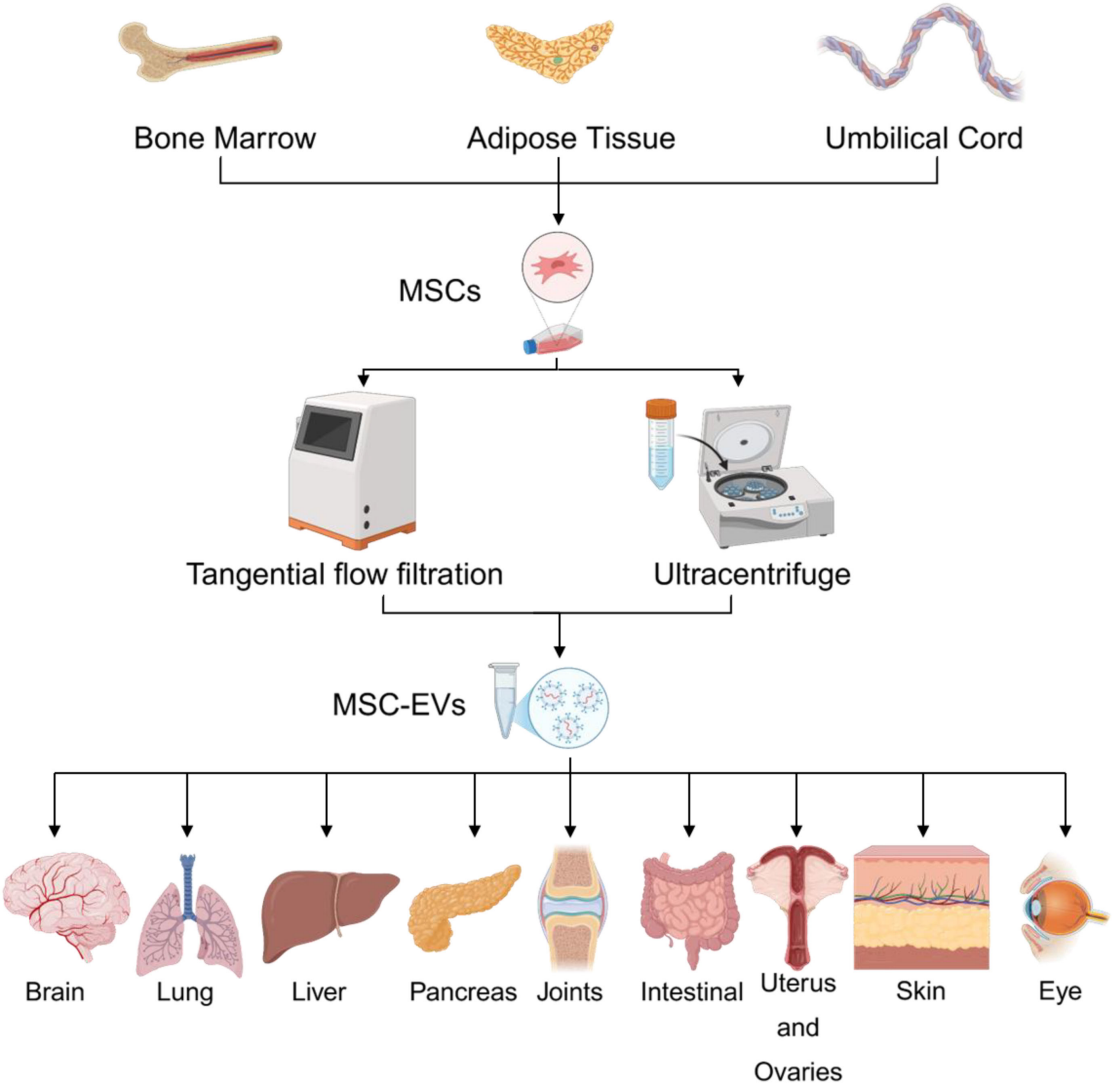
Summary of the sources, isolation methods, and organ-specific applications of mesenchymal stem cell-derived extracellular vesicles.

Screening Process: A total of 187 clinical trials were retrieved. After eliminating 39 duplicate trials and excluding 38 trials through preliminary screening of titles and abstracts, the research data of 90 clinical trials were evaluated against the inclusion criteria. Finally, 66 clinical trials were included in the analysis. The screening process is illustrated in Figure 2, and an overview is shown in Figure 1. The generated dataset is presented in Supplementary Appendix Table 1, which includes the baseline data of clinical trials of MSC-EVs and MSC-Exos, and Supplementary Appendix Table 2, which details the intervention methods of these.

### 3.2 Overview of clinical trial datasets

**FIGURE 3:**
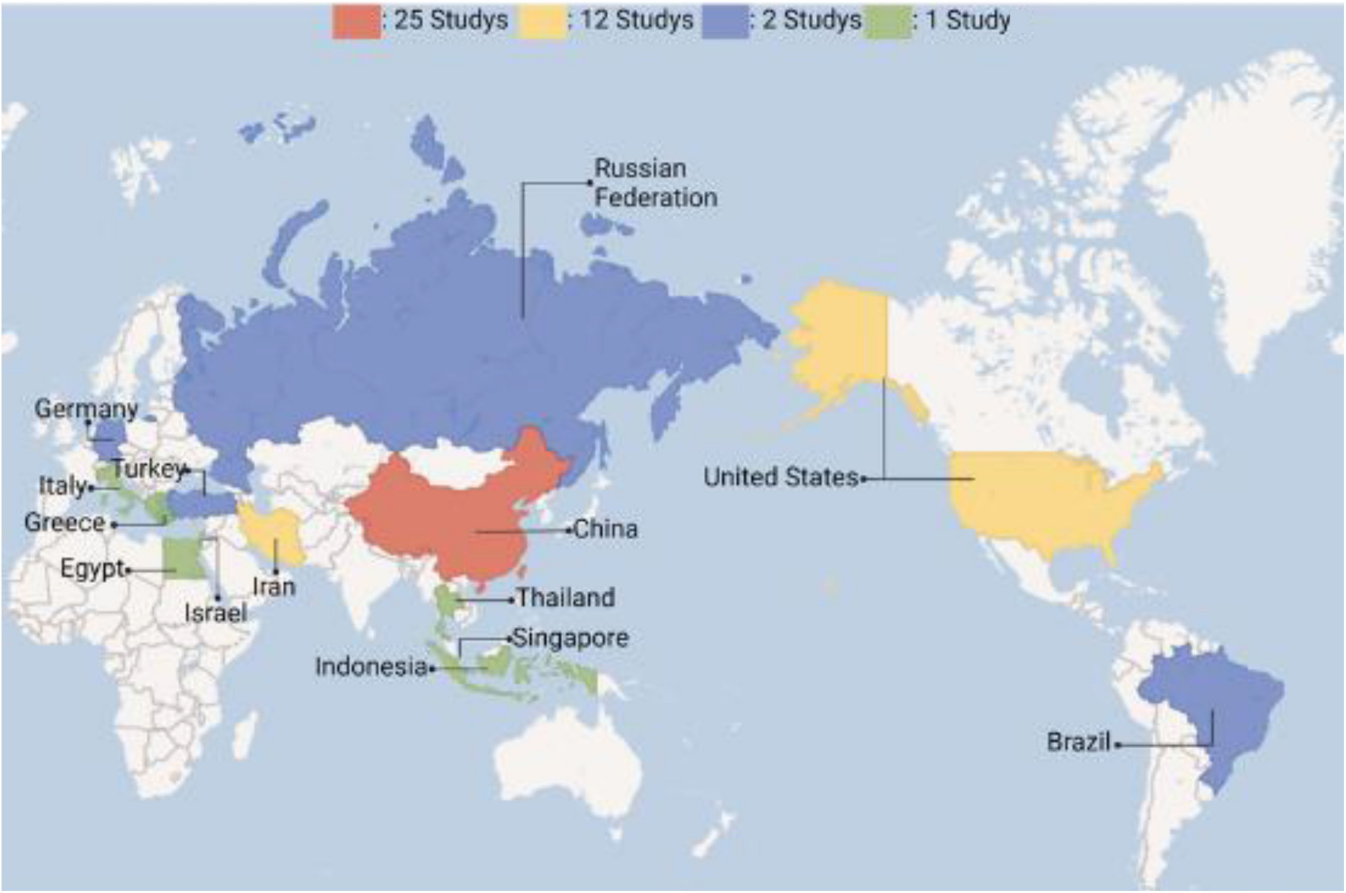
Number of countries and projects conducting relevant clinical trials worldwide.

**FIGURE 4:**
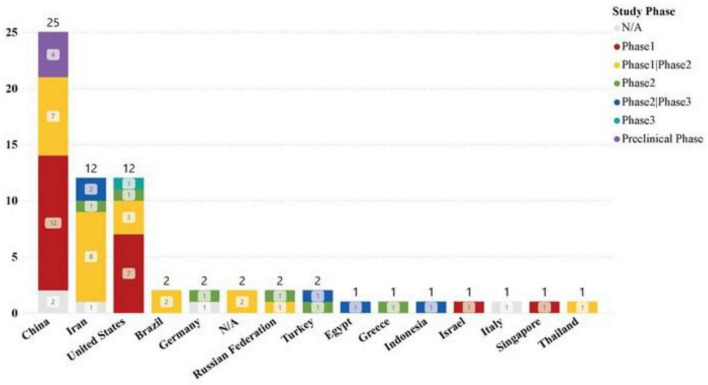
Clinical trials in different countries.

**FIGURE 5:**
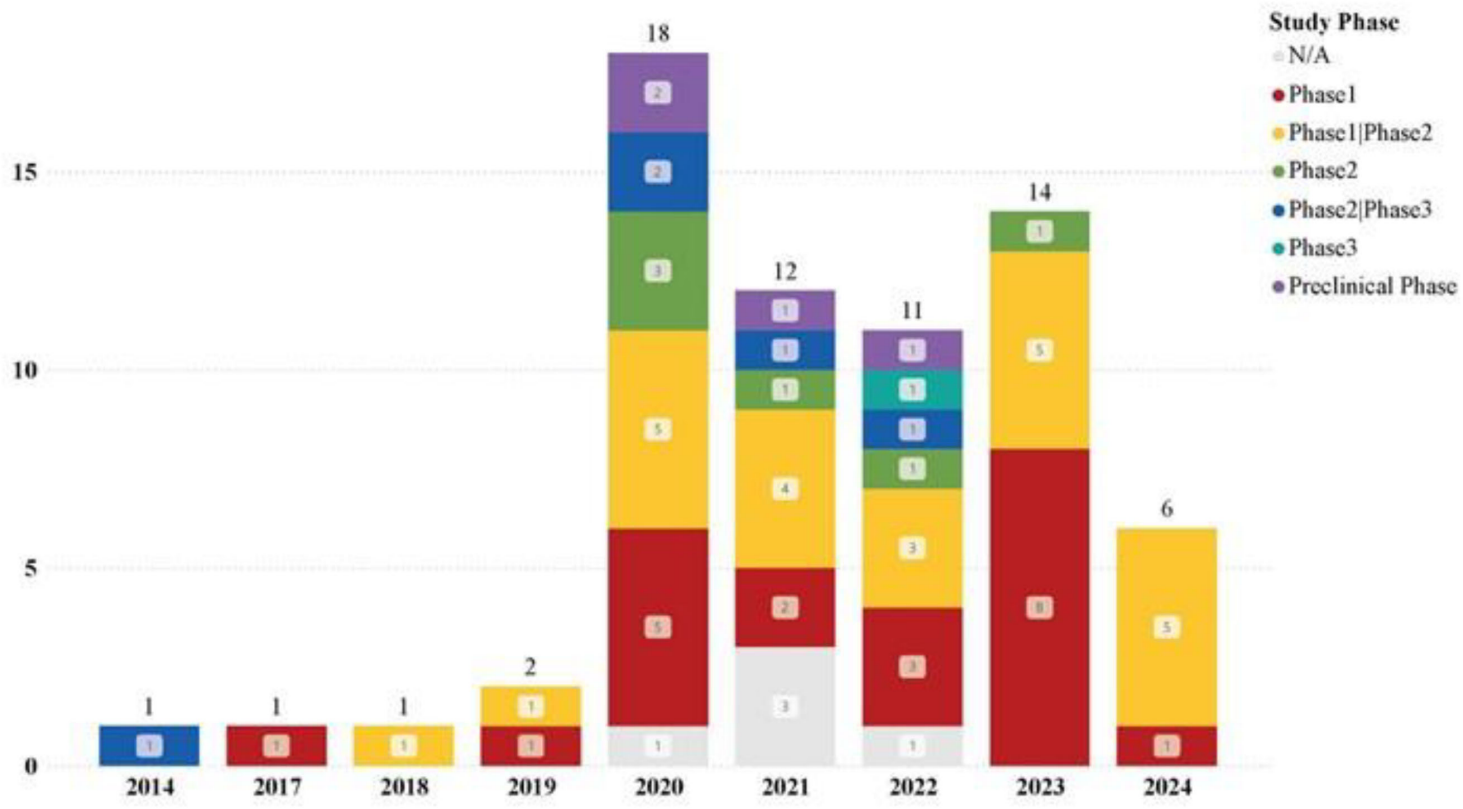
Trends in newly registered clinical trials on MSC-EVs and MSC-Exos at different stages each year.

The registration of clinical trials related to MSC-EVs and MSC-Exos has been ongoing from April 2014 to the present. The dataset included in this study covers a span of ten years. The distribution of countries/regions involved in these studies shows that China ranks first with 30 studies, followed by Iran and the United States, each with 12 studies, indicating high research activity. Other countries such as Turkey, Brazil, Germany, and the Russian Federation each have 2 studies, while Singapore, Egypt, Indonesia, Thailand, Italy, Greece, and Israel have only 1 study each, as shown in Figures 3, 4. The number of studies before 2020 was only 5, but 2020 marked a breakthrough, showing an explosive growth trend, including 26 studies on lung diseases and 23 clinical trials related to COVID-19. According to Medidata, the COVID-19 pandemic negatively impacted ongoing and newly launched clinical trials worldwide, particularly causing delays in patient enrollment and recruitment. However, clinical trials related to MSC-EVs and MSC-Exos have shown an upward trend and are actively used to explore treatments for COVID-19. The research phases of related clinical trials are predominantly preclinical, phase I, or early phase I and II studies, with 61 trials (92.24%) falling into these categories (Figure 5). Additionally, interventional studies dominate the research methods of clinical trials related to MSC-EVs and MSC-Exos, with 60 trials (90.91%) adopting this approach. The preferred research designs are parallel trials (33 trials, 50.00%) and single-arm trials (20 trials, 30.30%).

### 3.3 Analysis of MSCs tissue origin in clinical trial datasets

We made extensive efforts to collect the sources of MSCs from study titles, abstracts, designs, and intervention methods in the registration database through the MSC-EVs and MSC-Exos related clinical trial datasets. Despite supplementing missing sources in the records, there were still 22 studies that did not clearly mention the tissue sources of the MSCs used.

**FIGURE 6:**
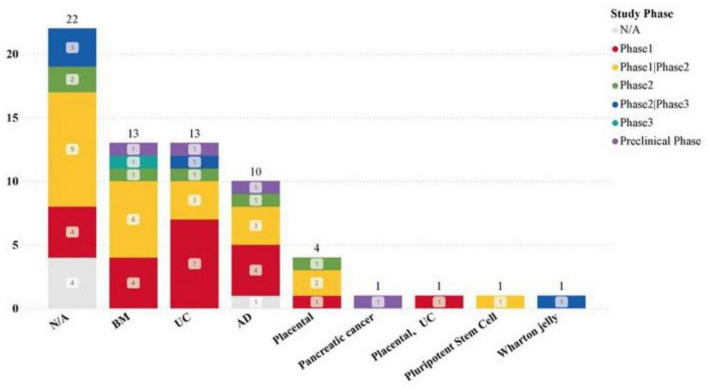
Application of MSCs from different tissues in clinical trials.

The clearly stated tissue sources in the dataset include: Umbilical cord, Bone marrow, Adipose tissue, Placenta, Wharton’s jelly, Pluripotent stem cells, Tumor stroma. Among these, umbilical cord (14 studies, 29.55%), bone marrow (13 studies, 29.55%), and adipose tissue (10 studies, 22.73%) are the most common sources, with all three having conducted phase III clinical trials (Figure 6). This trend may indicate that factors such as the difficulty of obtaining different tissue sources, immunogenicity, and proliferation rate have limited the development of clinical trials related to MSCs from other tissues. Additionally, it suggests that an increasing number of clinical trials are using EVs and Exos extracted from commercially available MSCs cell banks.

### 3.4 Clinical trial dataset disease and injury application analysis

Our dataset examined the specificity of diseases in clinical trials, revealing a significant focus on pulmonary disease (26 studies) and a high number of COVID-19-related clinical trials (23 studies). Additionally, skin, eye, anorectal, bone, and joint diseases have also been widely studied.

**FIGURE 7:**
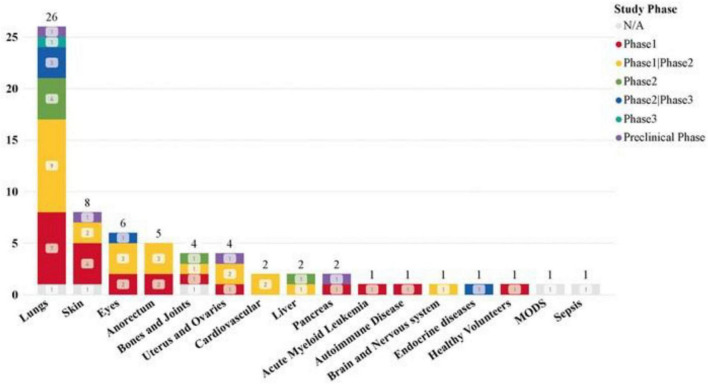
Clinical trials of MSC-EVs and MSC-Exos in different diseases and injuries.

**FIGURE 8:**
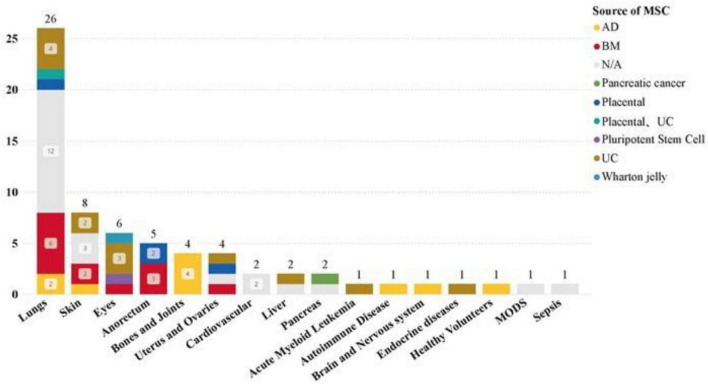
Distribution of MSCs from different tissues in different disease types in clinical trials.

The most common drug administration methods for lung diseases are: intravenous infusion (15 studies), aerosolization (12 studies). Statistics on the sources of MSCs used in different disease types show that in the treatment of lung diseases, MSC-EVs and MSC-Exos derived from bone marrow (BM) and umbilical cord (UC) tissues are significant research hotspots (Figures 7, 8). This trend highlights the relevance and potential of BM and UC-derived MSC-EVs and MSC-Exos in treating lung diseases, particularly in the context of the COVID-19 pandemic. The detailed analysis of the dataset underscores the evolving focus on specific MSCs sources and administration methods to optimize therapeutic outcomes in various disease contexts.

### 3.5 Drug administration route analysis of clinical trial datasets

The main routes of administration for clinical studies of MSC-EVs and MSC-Exos are intravenous infusion, nebulization, and local administration. The dataset includes the following: intravenous injection (24 studies), local administration (15 studies), nebulization (13 studies).

**FIGURE 9:**
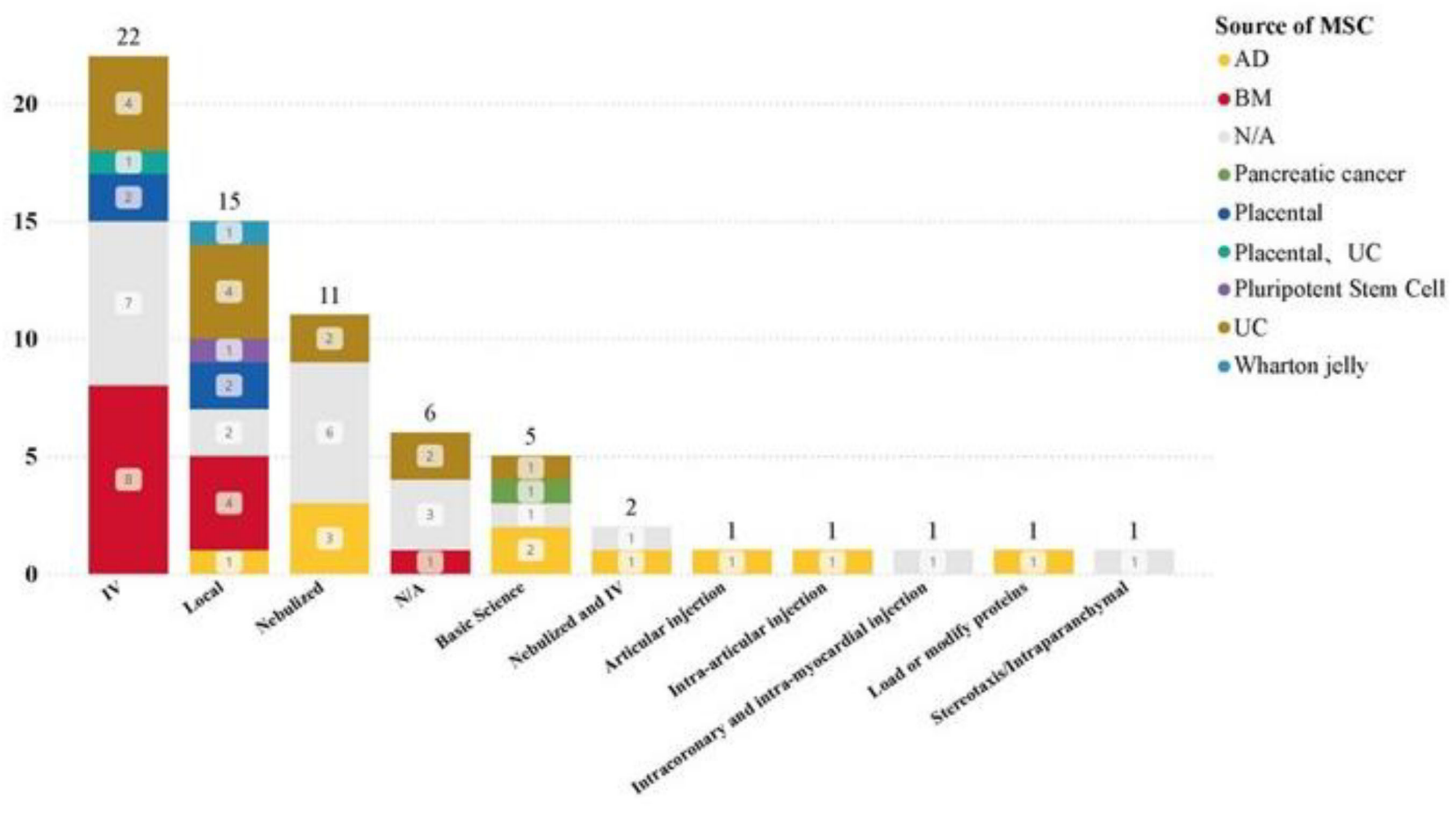
Administration of MSC-EVs and MSC-Exos in different disease types.

Analysis of the distribution of clinical trial research stages reveals that intravenous infusion and nebulization cover the widest range of clinical trial stages. Both methods have advanced to clinical phase III trials, indicating that intravenous infusion and nebulization are at the forefront of clinical research and are relatively mature methods (see Figure 9). This analysis highlights the prominence and progress of intravenous infusion and nebulization as preferred administration routes in the clinical study of MSC-EVs and MSC-Exos. Their maturity and widespread application across different clinical trial stages underscore their potential for broader clinical adoption and therapeutic use.

## 4 Dose-effect analysis of clinical trial datasets

Given the wide range of doses, we attempted to confirm whether there is an optimal dose range for MSC-EVs and MSC-Exos. We screened out six clinical trials with reported results and included them in the final dose-effect comprehensive analysis. The statistical data focused on dose, frequency of administration, administration method, and statistical descriptions and indicators of the effects in the reported results. After integrating the relevant data, Table 2 were obtained.

The results indicated that the disease specificity of clinical trials with relevant results was predominantly lung disease. However, due to differences in the unit mode of MSC-EVs or MSC-Exos in dose calculation-some studies used microgram weight, some calculated by the number of particles, and others stated the number of MSCs used for extraction-a unified standard was adopted as much as possible. The effect indicators were statistically summarized and comprehensively evaluated based on tolerability, safety, adverse events, and dose-effect trends.

The 6 clinical trials included in the dose-effect analysis were divided according to the route of administration: Nebulized inhalation: 3 studies, Intravenous infusions: 2 studies.

Intravenous infusion of MSCs combined with nebulized inhalation of MSC-EVs: 1 study.

Nebulized Inhalation Dose Tolerance and Efficacy: In the study on healthy subjects’ tolerability (NCT04313647) (13), a single dose of 2∼16 × 10^8^ particles of ATMSC-EVs was well tolerated in the short term without adverse reactions. In a pilot study (NCT04276987) (14) involving 7 patients with severe COVID-19-related pneumonia inhaling 2 × 10^8^ particles of ATMSC-Exos for 5 consecutive days, all patients tolerated it well with no evidence of pre-specified adverse events or clinical instability. Slight increases in lymphocyte counts and varying degrees of regression in lung lesions evaluated by CT were observed. Another study (NCT04491240) (15) with a dose range of 0.5∼2 × 10^10^ particles, twice a day for 10 consecutive days, showed significant improvements in inflammatory indicators such as C-reactive protein in patients with COVID-19-related pneumonia.

Intravenous Infusion and Combined Administration: In a study involving the simultaneous application of intravenous infusion of MSCs and aerosol inhalation of EVs (IRCT20200217046526N2) (16), no significant difference in outcome indicators was observed between using MSCs alone or in combination with EVs. However, the study showed that aerosol administration of MSC-EVs has good feasibility and efficacy comparable to cell therapy. The ExoFlo™ product (BMMSC-EVs) was used in two studies. A single-center study reported that 24 patients received a single 15 mL intravenous dose of ExoFlo™ (17). The patients’ clinical status, oxygenation, and laboratory indicators improved, with a survival rate of 83% and no adverse events observed within 72 h. In a further ongoing Phase II clinical trial (NCT04493242) (18), 102 patients were randomized into three groups receiving placebo, 10 mL ExoFlo™, or 15 mL ExoFlo™ on days 1 and 4. No treatment-related adverse events were reported. The 60-day overall survival rate was 51.6% in the placebo group, 53.4% in the 10 mL group, and 69.6% in the 15 mL group. A dose-effect trend was observed for ventilator-free days (VFDs): 32.0 ± 26.23 in the 10 mL group and 41.3 ± 25.75 in the 15 mL group. In a study on bronchial dysplasia treated with intravenous infusion, the trial was terminated due to fatal serious adverse events in the lowest dose group (20 Pmol/kg): necrotizing colitis occurred in 1 out of 2 patients (50%) (19).

Based on the above results, the analysis focused on individual trials reporting at least one effective dose or one less effective dose. The results showed that compared to nebulized inhalation, the intravenous infusion route required a higher dose to reach the effective range. Under the dose intervention conditions, there was a dose-effect trend, suggesting that doses lower or higher than the minimum effective dose (MED) were not ideal. This indicates a relatively narrow optimal effective dose range in the dose-effect relationship.

## 5 Discussion

### 5.1 Current clinical landscape and research priorities

This study retrieved data from three major databases, identifying 187 global clinical trials involving MSC-derived EVs and Exos. After applying inclusion and exclusion criteria, 66 relevant trials were included, resulting in two research datasets (see Supplementary Appendix Tables 1, 2). These datasets systematically report on clinical trials related to MSC-EVs and MSC-Exos, with visual analysis highlighting research focus areas across different regions from 2014 to 2024. The analysis reveals preferences in intervention methods (60 trials), phase I/II stages (61 trials), parallel control (33 trials), and single-arm designs (20 trials). Additionally, it identifies research trends in nine administration routes and seven tissue sources. Disease-specific analysis indicates that lung disease (26 trials) is a current research hotspot, with aerosol inhalation being a key administration method. Consistent with the MISEV2018 guidelines published by the International Society for Extracellular Vesicles (ISEV), accurate terminology is essential for the rigorous reporting and interpretation of EV-based research. In this review, we use “extracellular vesicle (EV)” as the generic term and refer to “exosomes” only when the original study provides sufficient evidence of endosomal origin, such as through differential ultracentrifugation, specific marker profiling (e.g., CD63, CD81, CD9), or transmission electron microscopy. Given that most clinical trials do not perform detailed vesicle subtyping, the blanket use of “exosome” in early-phase clinical studies can be misleading. Standardized nomenclature not only supports scientific reproducibility and inter-study comparability but is also critical for regulatory clarity and the development of EV-specific guidelines for clinical use.

### 5.2 Dose–effect relationships and administration-specific considerations

Clinical trials with reported results predominantly focus on lung disease, displaying differences in effective dose magnitudes associated with intravenous infusion and aerosol inhalation, as well as a range of minimum effective doses (MEDs) in clinical applications. This dynamic and rapidly evolving field, particularly concerning lung diseases and COVID-19, has significant implications for reducing long-term costs, increasing the reporting of clinical trial results (including negative results), minimizing unnecessary trial duplications, and avoiding ineffective doses.

In-depth exploration of these findings reveals potential challenges and future directions. The rapid response to the COVID-19 pandemic in exploring MSC-EVs and Exos for therapeutic purposes is both timely and crucial (20). These efforts not only address current challenges but also set a precedent for rapid scientific mobilization in future global health crises (21). The preference for intravenous and aerosol routes underscores the strategic focus on maximizing therapeutic efficacy, particularly for respiratory diseases, while local administration may enhance therapeutic effects and minimize systemic exposure (22–24).

The diversity of MSCs sources, particularly umbilical cord, bone marrow, and adipose tissue, highlights the need for a broader understanding of tissue-source specificity and its therapeutic implications (25). Diversifying tissue sources may facilitate more personalized and effective treatments tailored to individual patients or specific disease mechanisms. To fully exploit the therapeutic potential of MSC-EVs and Exos, a deeper understanding of their mechanisms of action is essential (9). The variation in dose calculation units and lack of standardized protocols pose significant challenges, complicating comparisons and meta-analyses. Establishing standardized indicators and ensuring consistency in preparation, characterization, and dose units across different studies is crucial for advancing clinical applications (26–28).

The dose-effect relationship of MSC-EVs and MSC-Exos is vital for optimizing therapeutic outcomes, ensuring safety, and minimizing adverse effects. This relationship is influenced by multiple factors, including the nature of the disease, route of administration, and patient-specific variables. Determining the optimal dose range is critical to maximizing therapeutic effects while minimizing potential risks. Experimental results indicate that the nature of the disease being treated plays a crucial role in determining the appropriate dose. Diseases localized to specific areas, such as wounds or lung diseases, may respond better to lower doses delivered directly to the affected area. In contrast, systemic diseases may require higher doses or targeted delivery strategies to achieve the desired therapeutic effect (29).

For lung diseases, aerosolization can deliver treatment directly to the lungs, potentially requiring lower doses compared to systemic administration routes such as intravenous infusion. This direct delivery can enhance local therapeutic effects while reducing systemic exposure and potential side effects (28, 30–32). The optimal dose range for MSC-EVs and MSC-Exos is narrow, particularly when administered by aerosolization or intravenous routes, emphasizing the importance of precise dosing in clinical applications. Doses that are too low may be ineffective, while doses that are too high may cause adverse reactions. Understanding the biological mechanisms of therapeutic effects can inform more targeted and effective dosing strategies, optimizing doses according to specific therapeutic targets or pathways.

### 5.3 Translational barriers, regulatory challenges, and future directions

Regulatory and ethical considerations—such as donor variability, batch-to-batch consistency, and long-term safety—are central to the clinical adoption of MSC-EVs and MSC-Exos. Promoting global collaboration and open data sharing can accelerate progress by pooling resources, expertise, and datasets, thereby avoiding duplication and facilitating faster translation into patient benefit. However, commercialization introduces additional challenges, including scaling up production, maintaining product quality, ensuring affordability, and securing equitable access. While commercial cell banks may mitigate some logistical issues, they also raise concerns regarding proprietary technologies, cost structures, and fair distribution.

Despite growing therapeutic promise, MSC-EVs translation remains constrained by persistent methodological, regulatory, and commercial barriers. Unlike conventional biologics or cell-based therapies, MSC-EVs lack standardized definitions for product identity, dosing units, and potency metrics—undermining reproducibility and delaying regulatory approval. Manufacturing heterogeneity, arising from differences in tissue source, donor characteristics, culture conditions, and isolation methods, further contributes to batch inconsistency. The absence of validated potency assays and pharmacokinetic/pharmacodynamic (PK/PD) data limits quality control and leaves uncertainties regarding biodistribution, clearance, and optimal dosing schedules. Cross-trial comparisons are hampered by inconsistent dose reporting (e.g., particle counts, protein content, volume) and reliance on unvalidated surrogate endpoints such as CRP or IL-6. To date, no MSC-EVs product has been approved by the FDA, EMA, or comparable authorities, reflecting a fragmented global regulatory framework for EV classification, GMP compliance, and clinical evaluation.

Compounding these issues is the uncontrolled proliferation of unregulated “exosome therapies” outside formal clinical frameworks, posing substantial risks to patient safety and eroding scientific credibility. Addressing these concerns requires internationally harmonized manufacturing, characterization, and reporting protocols; consensus-driven nomenclature (e.g., per MISEV guidelines); validated clinical endpoints; and regulatory convergence. Designing clinical trials with standardized dosing metrics, robust dose–response evaluation, and comprehensive adverse event reporting will be critical to defining optimal therapeutic windows across diseases and administration routes.

Ultimately, advancing MSC-EVs from experimental tools to evidence-based therapeutics will demand coordinated efforts from academia, industry, and regulatory agencies to establish rigorous standards, protect patient safety, and uphold scientific integrity. As the field evolves, integration of precision medicine approaches and patient-centered strategies will be pivotal to fully realizing the therapeutic potential of MSC-EVs in clinical practice.
