# Supplementary material for: Trends in mesenchymal stem cell-derived extracellular vesicles clinical trials 2014–2024: is efficacy optimal in a narrow dose range?
Source: Front Med (Lausanne). 2025 Sep 18;12:1625787. doi: 10.3389/fmed.2025.1625787 (PMC12488731; doi:10.3389/fmed.2025.1625787)
Supplement: Supplementary file 1 [file Table_1.docx]

Appendix 1. Baseline data of clinical trials of extracellular vesicles and exosomes derived from mesenchymal stem cells

| **Registration number** | **Start Date** | **Study type** | **Study Phase** | **Study design** | **Organ** | **Disease** | **Route of administration** | **Source of MSCs** |
| --- | --- | --- | --- | --- | --- | --- | --- | --- |
| NCT05808400/ChiCTR2300069181 | March-23 | Interventional | Phase1 | Non randomized control | Lungs | COVID-19 related Diseases | Nebulized | UC |
| ChiCTR2300067975 | March-23 | Interventional | Phase1 | Parallel | Skin | Skin Disease | Local | UC |
| ChiCTR2300067950 | February-23 | Interventional | Phase1 | Parallel | Skin | Skin Disease | Local | UC |
| ChiCTR2200061216 | June-22 | Basic Science | Preclinical Phase | Single group | Skin | Skin Photoaging | Load or modify proteins | AT |
| ChiCTR2200059351 | April-22 | Interventional | Phase1 | Case study | Bones and Joints | joint disease | Intra-articular injection | AT |
| ChiCTR2100050092 | Aμgust-21 | Basic Science | Preclinical Phase | Sequential | Pancreas | Pancreatic Disease | Basic Science | Pancreatic cancer |
| NCT04850469/ChiCTR2100044280 | March-21 | Interventional | N/A | Parallel | Sepsis | Sepsis | N/A | N/A |
| ChiCTR2100042948 | February-21 | Interventional | Phase1 | Non randomized control | Autoimmune Disease | Autoimmune Disease | Nebulized、IV | AT |
| NCT04602104/ChiCTR2000037250/ChiCTR2000035771 | Aμgust-20 | Interventional | Phase1\|Phase2 | Parallel | Lungs | ARDS | Nebulized | N/A |
| NCT04388982/ChiCTR2000032579 | May-20 | Interventional | Phase1\|Phase2 | Dose comparison | Brain and Nervous system | Alzheimer Disease | Local | AT |
| ChiCTR2000032537 | May-20 | Basic Science | Preclinical Phase | Sequential | Uterus and Ovaries | Reproductive system diseases | Basic Science | UC |
| ChiCTR2000031188 | March-20 | Interventional | Phase1 | Parallel | Eyes | Eye Disease | Local | UC |
| ChiCTR2000031122 | March-20 | Interventional | Preclinical Phase | Parallel | Lungs | Bronchial asthma | N/A | BM |
| ChiCTR2000030261 | February-20 | Interventional | Phase1 | Parallel | Lungs | COVID-19 | Nebulized | N/A |
| ChiCTR1800018327 | September-18 | Observational | Phase1\|Phase2 | Sequential | Uterus and Ovaries | Reproductive system diseases | N/A | N/A |
| NCT05658094 | Aμgust-22 | Interventional | N/A | Single group | Skin | Alopecia | N/A | N/A |
| NCT05813379 | February-22 | Interventional | Phase1\|Phase2 | Single group | Skin | Skin Anti Aging | Local | N/A |
| NCT05871463 | May-23 | Interventional | Phase2 | Single group | Liver | Liver Disease | N/A | UC |
| NCT04173650 | January-24 | Interventional | Phase1\|Phase2 | Single group | Skin | Skin Disease | Local | BM |
| NCT04544215 | July-20 | Interventional | Phase1\|Phase2 | Parallel | Lungs | bacterial pneumonia | Nebulized | AT |
| NCT05387278 | March-23 | Interventional | Phase1 | Parallel | Lungs | COVID-19 related Diseases/ARDS | IV | Placental、UC |
| NCT05523011 | March-22 | Interventional | Phase1 | Single group | Skin | Skin Disease | Local | N/A |
| NCT03437759 | March-17 | Interventional | Phase1 | Parallel | Eyes | Eye Disease | N/A | UC |
| NCT05499156/IRCT20200413047063N3 | May-22 | Interventional | Phase1\|Phase2 | Parallel | Anorectum | Anorectal diseases | Local | Placental |
| NCT04493242 | September-20 | Interventional | Phase2 | Parallel | Lungs | COVID-19/ARDS | IV | BM |
| NCT05787288 | January-23 | Interventional | Phase1 | Parallel | Lungs | COVID-19 | Nebulized | UC |
| NCT02138331 | April-14 | Interventional | Phase2\|Phase3 | Single group | Endocrine diseases | Type 1 diabetes | IV | UC |
| NCT04798716 | September-23 | Interventional | Phase1\|Phase2 | Sequential | Lungs | COVID-19 | iv | N/A |
| NCT05261360 | March-22 | Interventional | Phase2 | Parallel | Bones and Joints | joint disease | Articular injection | AT |
| NCT04213248 | February-20 | Interventional | Phase1\|Phase2 | Single group | Eyes | Eye Disease | Local | UC |
| NCT05402748/IRCT20211212053361N1 | December-21 | Interventional | Phase1\|Phase2 | Parallel | Anorectum | Anorectal diseases | Local | Placental |
| NCT05413148 | Aμgust-22 | Interventional | Phase2\|Phase3 | Parallel | Eyes | Eye Disease | Local | Wharton jelly |
| NCT04313647 | March-20 | Interventional | Phase1 | Parallel | Healthy Volunteers | Healthy Volunteers | Nebulized | AT |
| NCT04356300 | September-20 | Interventional | N/A | Parallel | MODS | MODS | IV | N/A |
| NCT05738629 | March-24 | Interventional | Phase1\|Phase2 | Single group | Eyes | Eye Disease | Local | Pluripotent Stem Cell |
| NCT04998058 | December-23 | Interventional | Phase1\|Phase2 | Parallel | Bones and Joints | Bone Loss | Basic Science | AT |
| NCT05354141 | July-22 | Interventional | Phase3 | Parallel | Lungs | ARDS | IV | BM |
| NCT03384433 | April-19 | Interventional | Phase1\|Phase2 | Single group | Cardiovascular | cerebrovascular disease | Stereotaxis/Intraparanchymal | N/A |
| NCT04276987 | February-20 | Interventional | Phase1 | Single group | Lungs | COVID-19 | Nebulized | AT |
| NCT06245746 | January-24 | Interventional | Phase1 | Single group | Acute Myeloid Leukemia | Acute Myeloid Leukemia | IV | UC |
| NCT05216562 | July-21 | Interventional | Phase2\|Phase3 | Parallel | Lungs | COVID-19 | IV | N/A |
| NCT03608631 | January-21 | Interventional | Phase1 | Single group | Pancreas | Pancreatic Disease | IV | N/A |
| NCT06072794 | October-23 | Interventional | Phase1 | Single group | Uterus and Ovaries | Reproductive system diseases | IV | Placental |
| NCT05669144 | April-22 | Interventional | Phase1\|Phase2 | Parallel | Cardiovascular | Myocardial Infarction | Intracoronary and intra-myocardial injection | N/A |
| NCT05191381 | December-21 | Observational | N/A | Time perspective | Lungs | COVID-19 | Basic Science | N/A |
| NCT06242379 | January-24 | Interventional | Phase1\|Phase2 | Single group | Eyes | Eye Disease | IV | BM |
| NCT05881668/NCT05940610 | May-23 | Interventional | Phase1\|Phase2 | Parallel | Liver | Liver Disease | IV | N/A |
| NCT05836883 | Aμgust-23 | Interventional | Phase1\|Phase2 | Sequential | Anorectum | Anorectal diseases | Local | BM |
| NCT05130983 | January-23 | Interventional | Phase1 | Single group | Anorectum | Anorectal diseases | iv | BM |
| NCT06002841 | February-24 | Interventional | Phase1\|Phase2 | Parallel | Lungs | ARDS | IV | N/A |
| NCT05176366 | December-22 | Interventional | Phase1 | Single group | Anorectum | Anorectal diseases | IV | BM |
| NCT05078385 | Aμgust-23 | Interventional | Phase1 | Single group | Skin | Burns | Local | BM |
| NCT05127122 | November-21 | Interventional | Phase1\|Phase2 | Crossover | Lungs | ARDS | IV | BM |
| NCT04223622 | April-21 | Observational | N/A | Time perspective | Bones and Joints | joint disease | Basic Science | AT |
| NCT03857841 | February-19 | Interventional | Phase1 | Sequential | Lungs | Bronchopulmonary Dysplasia | IV | BM |
| NCT06202547/IRCT20080831001141N43 | December-23 | Interventional | Phase1\|Phase2 | Single group | Uterus and Ovaries | Reproductive system diseases | Local | BM |
| NCT05116761 | November-24 | Interventional | Phase1\|Phase2 | Parallel | Lungs | COVID-19 related Diseases | IV | BM |
| NCT04902183 | June-21 | Interventional | Phase2 | Parallel | Lungs | COVID-19 | Nebulized | N/A |
| NCT04602442 | October-20 | Interventional | Phase2 | Parallel | Lungs | COVID-19 | Nebulized | N/A |
| NCT04747574 | September-20 | Interventional | Phase1 | Single group | Lungs | COVID-19 | Nebulized | N/A |
| NCT04491240 | July-20 | Interventional | Phase1\|Phase2 | Parallel | Lungs | COVID-19 | Nebulized | N/A |
| NCT04366063/IRCT20200217046526N2 | April-20 | Interventional | Phase2\|Phase3 | Parallel | Lungs | COVID-19 | IV | N/A |
| ISRCTN33578935 | June-20 | Interventional | Phase2 | Parallel | Lungs | COVID-19 | IV | Placental |
| IRCT20190101042197N2 | March-21 | Interventional | Phase1\|Phase2 | Parallel | Lungs | COVID-19 | IV | UC |
| IRCT20200217046526N2 | April-20 | Interventional | Phase2\|Phase3 | Parallel | Lungs | COVID-19 | Nebulized、IV | N/A |
| IRCT20201202049568N3 | March-21 | Interventional | Phase1\|Phase2 | Parallel | Lungs | COVID-19 | IV | UC |

Appendix 2. Intervention methods and results of clinical trials of mesenchymal stem cell-derived extracellular vesicles and exosomes

| **Registration number** | **Study Title** | **Country** | **Description for medicine or protocol of treatment in detail** | **Study Results** | **STUDY URL** |
| --- | --- | --- | --- | --- | --- |
| NCT05808400/ChiCTR2300069181 | A Clinical study on the safety and efficacy of umbilical cord mesenchymal stem cell exosomes in treating chronic coμgh with post-COVID-19 syndrome | China | Exosomes from umbilical cord mesenchymal stem cells (5ml, exosome concentration was 1 x 10^9^ Particles /ml; Protocal of treament: Nebulized Inhalation for 5 days, Bid. | N | <https://www.chictr.org.cn/showproj.html?proj=191614> |
|  |  |  |  |  | <https://clinicaltrials.gov/study/NCT05808400> |
| ChiCTR2300067975 | Clinical observation on treatment of vitiligo with exosomes of umbilical cord mesenchymal stem cell | China | Topical exosomes gel | N | <https://www.chictr.org.cn/showproj.html?proj=187006> |
| ChiCTR2300067950 | Clinical observation on treatment of psoriasis with exosomes of umbilical cord mesenchymal stem cells | China | Topical exosomes gel | N | <https://www.chictr.org.cn/showproj.html?proj=187008> |
| ChiCTR2200061216 | Application of circCOL-ELNs carried by exosomes of adipose derived stem cells in the intervention of skin photoaging | China | N/A | N | <https://www.chictr.org.cn/showproj.html?proj=171319> |
| ChiCTR2200059351 | Phase I Study: Evaluating the Safety and feasibility of Autologous, Culture-expanded Adipose Mesenchymal Stem Cells-derived Exosomes in subjects with osteoarthritis | China | Articular injection of adipose tissue mscs-derived exosome 2ml/4m/6mll | N | <https://www.chictr.org.cn/showproj.html?proj=166361> |
| ChiCTR2100050092 | Effect of exosomes from carcinoma-associated mesenchymal stem cells on ferroptosis in pancreatic cancer cells and its mechanism | China | N/A | N | <https://www.chictr.org.cn/showproj.html?proj=130942> |
| NCT04850469/ChiCTR2100044280 | Clinical trial of exosomes derived from mesenchymal stem cells on the therapy for children with severe infection | China | N/A | N | <https://www.chictr.org.cn/showproj.html?proj=123326> |
|  |  |  |  |  | <https://clinicaltrials.gov/study/NCT04850469> |
| ChiCTR2100042948 | the safety and the efficacy study of allogenic adipose MSC-Exos in the treatment of anti-MDA5 positive dermatonyositis- associated interstitial lung disease | China | Routine treatment combined with aerosol inhalation of allogenic adipose MSC-Exos group  outine treatment combined with intravenous injection of allogenic adipose MSC-Exos group  routine treatment combined with aerosol inhalation and intravenous injection of allogenic adipose MSC-Exos group outine treatment | N | <https://www.chictr.org.cn/showproj.html?proj=121212> |
| NCT04602104/ChiCTR2000037250/ChiCTR2000035771 | A Phase I Clinical Study on the Safety and Efficacy of Allogeneic Human Adipose-derived Mesenchymal Stem Cell Exosomes (haMSC-Exos) Nebulized Inhalation in the Treatment of Moderate to Severe Acute Respiratory Distress Syndrome (ARDS) | China | Phase 1: 7 times aerosol inhalation of hmsc-Exos (2、8、16*10^8^ Particles at Day 1, Day 2, Day 3, Day 4, Day 5, Day 6, Day 7)  Phase 2: basic treatment and 7 times aerosol inhalation of hmsc-Exos (a quarter of MTD/day、MTD/day、normal saline at Day 1, Day 2, Day 3, Day 4, Day 5, Day 6, Day 7) | N | <https://www.chictr.org.cn/showproj.html?proj=60337> |
|  |  |  |  |  | <https://www.chictr.org.cn/showproj.html?proj=58484> |
|  |  |  |  |  | <https://clinicaltrials.gov/study/NCT04602104> |
| NCT04388982/ChiCTR2000032579 | Open-Label, Single-Center, Phase I/Ⅱ Clinical Trial to Evaluate the Safety and the Efficacy of Exosomes Derived From Allogenic Adipose Mesenchymal Stem Cells (Allogenic Adipose MSC-Exos) in Patients with Mild to Moderate Dementia Due to Alzheimer's Disease | China | 5、10、20 μg mscs-Exos administrated for nasal drip | N | <https://www.chictr.org.cn/showproj.html?proj=51939> |
|  |  |  |  |  | <https://clinicaltrials.gov/study/NCT04388982> |
| ChiCTR2000032537 | Study for the mechanism of umbilical cord mesenchymal stem cell exosomes in the endometrial injury repair | China | N/A | N | <https://www.chictr.org.cn/showproj.html?proj=53151> |
| ChiCTR2000031188 | Application of human umbilical cord mesenchymal stem cells derived exosomes in the treatment of patients with dry eye disease | China | N/A | N | <https://www.chictr.org.cn/showproj.html?proj=51452> |
| ChiCTR2000031122 | Role of macrophage in immuno-modulation by mesenchymal stem cell derived exosome in asthma | China | N/A | N | <https://www.chictr.org.cn/showproj.html?proj=51355> |
| ChiCTR2000030261 | A study for the key technology of mesenchymal stem cells exosomes atomization in the treatment of novel coronavirus pneumonia (COVID-19) | China | N/A | N | <https://www.chictr.org.cn/showproj.html?proj=49963> |
| ChiCTR1800018327 | A clinical study for mesenchymal stem cells exosomes for treating uterine endometrial injury | China | N/A | N | <https://www.chictr.org.cn/showproj.html?proj=30580> |
| NCT05658094 | Exosome Effect on Prevention of Hairloss | Iran | Exosome (100e10 particle) injections with an interval of 14 days during two months.Each patients will receive 4 injection with an interval of 14 days during two months | N | <https://clinicaltrials.gov/study/NCT05658094> |
| NCT05813379 | Mesenchymal Stem Cells Derived Exosomes in Skin Rejuvenation | Iran | The Exosome will be injected into each standardized injection point in a superficial manner. The injections points are along the inferior border of cheek and mid-cheek and temple, where will be followed by 10-15 minutes of icing | N | <https://clinicaltrials.gov/study/NCT05813379> |
| NCT05871463 | Effect of Mesenchymal Stem Cells-derived Exosomes in Decompensated Liver Cirrhosis | Iran | Patients will receive standard medication plus MSC-derived exosomes at a final dose of 40mg in three weeks. Standard medication includes: a) treatment of the underlying cause of cirrhosis such as drμg treatment of hepatitis B and C. B) symptomatic treatment of port complications such as ascites, prevention of variceal bleeding, treatment and prevention of hepatic encephalopathy. | N | <https://clinicaltrials.gov/study/NCT05871463> |
| NCT04173650 | MSC EVs in Dystrophic Epidermolysis Bullosa | America | Ligible subjects will undergo a one-month observation period to confirm that the targeted wound is chronic (only single wounds with evidence of less than 20% closure over that period will be eligible for treatment). Once this has been established, up to 6 administrations of bm-msc evs will occur, at each to be given over a period of no more than 3 months. A maximum of 50 cm2 in total wound surface will be treated, and each administration will occur 14 days (+/- 7 days) but no less than 7 days apart. If the wound closes prior to 6 administrations, no additional doses will be given. Wound closure will be determined by complete re-epithelialization that is not subject to re-injury during dressing changes or as a result of normal daily activities (e.g. Wearing clothing, eating, sleeping). After the 6 doses of bm-msc evs are given, the wound will be followed monthly for a period of 4 months to the termination of the study at 8 months or, in the event the wound closes before receiving all 6 doses, for 4 month after the wound closes. | N | <https://clinicaltrials.gov/study/NCT04173650> |
| NCT04544215 | A Clinical Study of Mesenchymal Progenitor Cell Exosomes Nebulizer for the Treatment of Pulmonary Infection | China | Dosage 1 、2 of mpcs-derived exosomes  7 times aerosol inhalation of mpcs-derived exosomes (8.0、16*10^8^ nano vesicles/3 ml at Day 1, Day 2, Day 3, Day 4, Day 5, Day 6, Day 7  )No aerosol inhalation of mpcs-derived exosomes | N | <https://clinicaltrials.gov/study/NCT04544215> |
| NCT05387278 | Safety and Effectiveness of Placental Derived Exosomes and Umbilical Cord Mesenchymal Stem Cells in Moderate to Severe Acute Respiratory Distress Syndrome (ARDS) Associated With the Novel Corona Virus Infection (COVID-19) | America | EV-Pure™ and WJ-Pure™  The treatment consists of administration of WJ-Pure™ and EV-Pure™ plus standard care | N | <https://clinicaltrials.gov/study/NCT05387278> |
| NCT05523011 | Safety and Tolerability Study of MSC Exosome Ointment | Singapore | 100 µg MSC exosomes/g ointment. The subjects will apply exosome ointment along with Vesiderm liposome cream (TID per day, 20 days). Each day (from Day 1 to 20), the study product will be applied with a gap of 4 hours between three doses to a healthy area (one hand area) on the forearm using 1 fingertip unit (FTU). The Vesiderm liposome cream is meant to keep the application site moisturized. | N | <https://clinicaltrials.gov/study/NCT05523011> |
| NCT03437759 | MSC-Exos Promote Healing of MHs | China | Add treatment of exosomes derived from mesenchymal stem cells (MSC-Exo) after pars plana vitrectomy(PPV) and ILM peeling. | N | <https://clinicaltrials.gov/study/NCT03437759> |
| NCT05499156/IRCT20200413047063N3 | Safety of Injection of Placental Mesenchymal Stem Cell Derived Exosomes for Treatment of Resistant Perianal Fistula in Crohn's Patients | Iran | The procedure was undertaken in the operation room, and the patients were NPO 6 hours before the operation with intravenous sedation and oxygen supplementation with a mask. The patients, placed were placed in a lithotomy position. The external fistula opening was inspected, the tract was palpated, and using an Eisenhammer Retractor, the internal orifice was exposed seen. The lot was irrigated with saline several times using a small catheter to clear the pus and fecal material. After irrigation, a flexible fistula probe was inserted into the tract. Using the probe as a guide, we injected 5 ml of exosome solution into the tissue surrounding the lot. The injection depth was about 2about 2-3 mm of the soft tissue and sphincters of the anus. After the injection, the tract probe was extracted, the patients were under observation in the operation recovery rooming room recovery for 3 hours, and vital signs were monitored. The patients were then transferred to the surgery ward and were observed for 48 hours. The patients were again examined under sedation in the operation room for further evaluation six months after injection. | N | <https://clinicaltrials.gov/study/NCT05499156> |
| NCT04493242 | Extracellular Vesicle Infusion Treatment for COVID-19 Associated ARDS | America | Placebo Comparator: Placebo  Normal saline 100 ml  Experimental: Experimental Dose 1:Normal saline 90 ml and exoflo 10 ml  Experimental: Experimental Dose 2:Normal saline 85 ml and exoflo 15 ml | Y | <https://clinicaltrials.gov/study/NCT04493242> |
| NCT05787288 | A Clinical Study on Safety and Effectiveness of Mesenchymal Stem Cell Exosomes for the Treatment of COVID-19. | China | Umbilical cord mesenchymal stem cell-derived extracellular vesicle preparation、saline solution; Specification: 5ml, with extracellular vesicle concentration of 1 × 10^9^ Particles/ml in the preparation; | N | <https://clinicaltrials.gov/study/NCT05787288> |
| NCT02138331 | Effect of Microvesicles and Exosomes Therapy on β-cell Mass in Type I Diabetes Mellitus (T1DM) | Egypt | The first dose will be purified exosomes, ranging between 40-180 nm, in a dose of the supernatant produced from (1.22-1.51)×10 (6)/kg/IV.(Characterization of exosomes:CD63, CD9, Alix, TSG 101, HSP 70).  - The second dose, after 7 days, will be the microvesicles, ranging between 180-1000 nm, in a dose of the supernatant produced from (1.22-1.51) × 10 (6)/kg/IV. | N | <https://clinicaltrials.gov/study/NCT02138331> |
| NCT04798716 | The Use of Exosomes for the Treatment of Acute Respiratory Distress Syndrome or Novel Coronavirus Pneumonia Caused by COVID-19 | America | First Cohort:Five patients will receive an escalating dose every other day for a period of 5 days, with a minimum of 24 hours between doses recorded. Dose escalation will begin at 2 x 10^9^ exosomes, Escalating dose 2 X 10^9^, 4 X 10^9^, 8 X 10^9^/ml.  Second Cohort:Five patients will receive an escalating dose every other day for a period of 5 days, with a minimum of 24 hours between doses recorded. Dose escalation will begin at 4 x 10^9^ exosomes. Escalating dose 8 X 10^9^, 4 X 10^9^, 8 X 10^9^ ml.  Third Cohort  Five patients will receive a treatment dose of 8 X 10^9^ exosomes every other day for a period of 5 days, with a minimum of 24 hours between doses recorded.Dosed 8 X 10^9^, 8 X 10^9^, 8 X 10^9^ ml.  Fourth Cohort:Randomized Cohort Up to 40 patients may be enrolled in this phase of the trial. For those receiving the placebo (~25%), 3 doses will be given over the 5 day period, dispensed from identical vials with physician and patient blinded. The full dose of 8 X 10^9^ exosomes will be given to 75% of the patients in 3 doses over the course of 5 days, with one dose occurring every other day. | N | <https://clinicaltrials.gov/study/NCT04798716> |
| NCT05261360 | Clinical Efficacy of Exosome in Degenerative Meniscal Injury | Turkey | SF-MSC-EX Treatment Group (Experimental group's left knees)  The left knee will receive 1 million cells/kg SF-MSC-EX (Synovial fluid mesenchymal stem cell-derived exosome) by intra-articular injection method.  Experimental: SF-MSC Treatment Group (Experimental group's right knees)  The right knee will receive 1 million cells/kg SF-MSC (Synovial fluid-derived mesenchymal stem cell) by intra-articular injection method. | N | <https://clinicaltrials.gov/study/NCT05261360> |
| NCT04213248 | Effect of UMSCs Derived Exosomes on Dry Eye in Patients With cGVHD | China | Participants will receive artificial tears for 2 weeks to get the normalized baseline, followed by UMSC-exo 1μg/drop, four times a day for 14 days. The follow-up visit will be 12 weeks. | N | <https://clinicaltrials.gov/study/NCT04213248> |
| NCT05402748/IRCT20211212053361N1 | Safety and Efficacy of Injection of Human Placenta Mesenchymal Stem Cells Derived Exosomes for Treatment of Complex Anal Fistula | Iran | Human Placenta Mesenchymal Stem Cells Derived Exosomes injected in fistula tract of patients with complex perianal Fistula in 3 weekly episodes and it's safety and efficacy was evaluated. | N | <https://clinicaltrials.gov/study/NCT05402748> |
| NCT05413148 | The Effect of Stem Cells and Stem Cell Exosomes on Visual Functions in Patients With Retinitis Pigmentosa | Turkey | The volunteers will be randomized into three. The 1st group will be the placebo group (45 volunteers) and 0.5 cc saline physiological saline will be applied to the subtenon space. 2nd group of patients (45 volunteers) will undergo a subtenon injection of Wharton gel-derived mesenchymal stem cell suspension. A suspension containing mesenchymal stem cell exosomes from Wharton jelly will be applied to the subtenon distance to the 3rd group of patients (45 volunteers). A single eye of each volunteer will be included. Informed consent will be obtained from the volunteers. | N | <https://clinicaltrials.gov/study/NCT05413148> |
| NCT04313647 | A Tolerance Clinical Study on Aerosol Inhalation of Mesenchymal Stem Cells Exosomes In Healthy Volunteers | China | MEXVT clinical trial is an open‐label, dose‐escalation pilot study in which five cohorts of healthy volunteers received increasing doses of hamsc‐evs administered as a single nebulization. There are three or six subjects per cohort, with patients in each cohort receiving either 2.0 × 10^8^ Particles (first cohort), 4.0 × 10^8^ Particles (second cohort), 8.0 × 10^8^ Particles (third cohort), 12.0 × 10^8^ Particles (fourth cohort) or 16.0 × 10^8^ Particles (fifth cohort). | Y | <https://clinicaltrials.gov/study/NCT04313647> |
| NCT04356300 | Exosome of Mesenchymal Stem Cells for Multiple Organ Dysfuntion Syndrome After Surgical Repaire of Acute Type A Aortic Dissection | China | Exosome of MSC at a dose of 150mg will be given intravenously to Patients in the exosome of MSC arm once a day for 14 times. | N | <https://clinicaltrials.gov/study/NCT04356300> |
| NCT05738629 | Safety and Efficacy of Pluripotent Stem Cell-derived Mesenchymal Stem Cell Exosome (PSC-MSC-Exo) Eye Drops Treatment for Dry Eye Diseases Post Refractive Surgery and Associated With Blepharospasm | China | Participants will receive artificial tears for 2 weeks to get the normalized baseline, followed by PSC-MSC-Exo eye drops 0.125 ml/single eye/one time, four times a day for 12 weeks. The follow-up visit will be 12 weeks since the administration of PSC-MSC-Exo eye drops. | N | <https://clinicaltrials.gov/study/NCT05738629> |
| NCT04998058 | Autogenous Mesenchymal Stem Cell Culture-Derived Signalling Molecules as Enhancers of Bone Formation in Bone Grafting | Brazil | The bony floor of the test maxillary sinus will be aμgmented with synthetic bone substitute (boneceramic™ 1-2 mm) mixed with 10 to 15 ml of CM (test). The control site will receive bone substitute with saline. Lateral windows in both sinuses will be then closed with a collagen membrane (Bio-Gide™). | N | <https://clinicaltrials.gov/study/NCT04998058> |
| NCT05354141 | Extracellular Vesicle Treatment for Acute Respiratory Distress Syndrome (ARDS) (EXTINGUISH ARDS) | America | Intravenous administration of bone marrow mesenchymal stem cell derived extracellular vesicles:Normal saline 85 ml and exoflo 15 ml | N | <https://clinicaltrials.gov/study/NCT05354141> |
| NCT03384433 | Allogenic Mesenchymal Stem Cell Derived Exosome in Patients With Acute Ischemic Stroke | Iran | Allogenic mesenchymal stem cells derived exosome enriched by mir-124 | N | <https://clinicaltrials.gov/study/NCT03384433> |
| NCT04276987 | A Pilot Clinical Study on Inhalation of Mesenchymal Stem Cells Exosomes Treating Severe Novel Coronavirus Pneumonia | China | 5 times aerosol inhalation of mscs-derived exosomes (2.0*10E8 nano vesicles/3 ml at Day 1, Day 2, Day 3, Day 4, Day 5) | Y | <https://clinicaltrials.gov/study/NCT04276987> |
| NCT06245746 | UCMSC-Exo for Chemotherapy-induced Myelosuppression in Acute Myeloid Leukemia | China | UCMSC-Exo will be infused intravenously, UCMSC-Exo will be preset with 3 escalation dose levels in single time infusion. | N | <https://clinicaltrials.gov/study/NCT06245746> |
| NCT05216562 | Efficacy and Safety of EXOSOME-MSC Therapy to Reduce Hyper-inflammation In Moderate COVID-19 Patients | Indonesia | The EXOSOME-MSC will be tested as adjuvant, on top of standard COVID-19 drμgs. It will be injected to participants via intravenous route twice, in day-1 and day-7 of 14 days of study participation. | N | <https://clinicaltrials.gov/study/NCT05216562> |
| NCT03608631 | iExosomes in Treating Participants With Metastatic Pancreas Cancer With KrasG12D Mutation | America | Participants receive mesenchymal stromal cells-derived exosomes with krasg12d sirna IV over 15-20 minutes on days 1, 4, and 10. Treatment repeats every 14 days for up to 3 courses in the absence of disease progression or unacceptable toxicity. Participants who respond may continue 3 additional courses. | N | <https://clinicaltrials.gov/study/NCT03608631> |
| NCT06072794 | A Proof of Concept Study to Evaluate Exosomes From Human Mesenchymal Stem Cells in Women With Premature Ovarian Insufficiency (POI) | America | This is a dose escalation study. The study participants are organized in groups of 3 where each participant will receive one study treatment. | N | <https://clinicaltrials.gov/study/NCT06072794> |
| NCT05669144 | Co-transplantation of Mesenchymal Stem Cell Derived Exosomes and Autologous Mitochondria for Patients Candidate for CABG Surgery | Iran | A) Intracoronary and intra-myocardial injection of exosomes (5 patients) 1 ml of exosomes containing 100 micrograms of exosomes  b) Intracoronary and intra-myocardial injection of mitochondria (5 patients) 1 ml of exosomes containing 10 million mitochondria  c) co-transplantation Intracoronary and intra-myocardial injection of exosomes and mitochondria (5 patients) 1 ml of exosomes containing 100 micrograms of exosomes and 1 ml of exosomes containing 10 million mitochondria  placebo: 1 ml of placebo solution | N | <https://clinicaltrials.gov/study/NCT05669144> |
| NCT05191381 | Immune Modulation by Exosomes in COVID-19 | Germany | Co-incubation of patient-derived whole blood samples with mesenchymal stem cell derived exosomes and read-out of biomarkers, RNA and immune phenotypes after 24h. | N | <https://clinicaltrials.gov/study/NCT05191381> |
| NCT06242379 | Safety and Efficacy of Stem Cell Small Extracellular Vesicles in Patients With Retinitis Pigmentosa | Thailand | Single intravitreal injection of GMP-compliant bone marrow mesenchymal stem cell-derived small extracellular vesicles (50 μg) for single eye | N | <https://clinicaltrials.gov/study/NCT06242379> |
| NCT05881668/NCT05940610 | MSC-EV in Acute-on-Chronic Liver Failure After Liver Transplantation | China | After liver transplantation, on the basis of postoperative standard treatment (anti-infection treatment, immunosuppressive treatment, nutritional support treatment, etc.), an additional injection of MSC-EV will be received between the 1st and 5th days after transplantationpatients participated in the experimental cohort will be infused with a single dose of 10 E10 MSC-EV Particles per 100ml, at an appropriate time during the first 1-5 days after transplantation. | N | <https://clinicaltrials.gov/study/NCT05881668> |
|  |  |  |  |  | <https://clinicaltrials.gov/study/NCT05940610> |
| NCT05836883 | Study of ExoFlo for the Treatment of Perianal Fistulas | America | Subjects will be randomized 2:1 Investigational Medicinal Product (IMP) to normal saline (NS) in 3 cohorts of 12 subjects as follows:  Cohort 1: Local injection of 15 ml of IMP or NS on Day 0 (8 IMP, 4 NS) Cohort 2: Local injection of 30 ml of IMP or NS on Day 0 (8 IMP, 4 NS) Cohort 3: Local injection of 30 ml of IMP or NS on Day 0 and Month 3 (8 IMP, 4 NS) | N | <https://clinicaltrials.gov/study/NCT05836883> |
| NCT05130983 | Study of ExoFlo for the Treatment of Medically Refractory Crohn's Disease | America | Experimental: 15ml at Day 0, 2, 4, 30 ml at Week 2, Week 6, and every 4 weeks after to week 46  IV administration of 15 ml study agent at Day 0, Day 2, Day 4 and 30 ml at Week 2, Week 6 and every 4 weeks thereafter to week 46 (total # doses = 15). | N | <https://clinicaltrials.gov/study/NCT05130983> |
| NCT06002841 | Extracellular Vesicles From Mesenchymal Cells in the Treatment of Acute Respiratory Failure | Brazil | EV group: will consist of 10 participants who will receive two infusions of 25 ml of the investigational product (Plasma-Lyte A solution containing evs obtained from mscs), intravenously, at intervals of 48 h.  Placebo group: will consist of 5 participants who will receive an equal volume of Plasma-Lyte A, intravenously, following the same schedule as the IV group: two infusions with an interval of 48 hours. | N | <https://clinicaltrials.gov/study/NCT06002841> |
| NCT05176366 | Study of ExoFlo for the Treatment of Medically Refractory Ulcerative Colitis | America | Study Product, Dose, Route, Regimen:  IV administration of 15 ml of study agent at Day 0, Day 2, Day 4,A and 30 ml at Week 2, Week 6 and every 4 weeks thereafter to week 46 (n=10), (total # doses = 15). | N | <https://clinicaltrials.gov/study/NCT05176366> |
| NCT05078385 | Safety of Extracellular Vesicles for Burn Wounds | America | This study is designed to examine the safety and efficacy, in 10 patients of administration of allogeneic MSC EV to deep second degree burn wounds. The dose level delivered will be approximately 1 X 107 EV Particles for each cm2 treated area. Wounds eligible for treatment will not exceed 600 cm2 per wound or collection of wounds in a defined anatomical area (e.g., arm, leg, chest) treated. Patients with 20% or greater TBSA in total (3700 cm2 in a 70 kg, 175 cm subject) will be ineligible to participate in the study. The first treatment will be administered within 48 hours of the burn injury. Two additional administrations of EV will be given approximately one week (day 5-7 post-injury) and two weeks after the first treatment (unless the wound is fully closed, in which case the patient will continue to be monitored at weekly intervals throμgh 5 weeks, then at 8, 12, 26, and 54 weeks). | N | <https://clinicaltrials.gov/study/NCT05078385> |
| NCT05127122 | Bone Marrow Mesenchymal Stem Cell Derived Extracellular Vesicles Infusion Treatment for ARDS | N/A | Experimental: 10ml/15/ml Bone Marrow Mesenchymal Stem Cell Derived Extracellular Vesicles | N | <https://clinicaltrials.gov/study/NCT05127122> |
| NCT04223622 | Effects of ASC Secretome on Human Osteochondral Explants | Italy | The osteochondral explants isolated from arthroplasty patients will be induced to an OA phenotype and treated with ASC secretome (either complete conditioned medium or extracellular vesicles) in order to investigate its therapeutic potential. | Y | <https://clinicaltrials.gov/study/NCT04223622> |
| NCT03857841 | A Safety Study of IV Stem Cell-derived Extracellular Vesicles (UNEX-42) in Preterm Neonates at High Risk for BPD | America | Experimental: 20\60\200 pmol phospholipid/kg body weight.UNEX-42 is a preparation of extracellular vesicles that are secreted from human bone marrow-derived mesenchymal stem cells suspended in phosphate-buffered saline. | Y | <https://clinicaltrials.gov/study/NCT03857841> |
| NCT06202547/IRCT20080831001141N43 | Intra-ovarian Injection of MSC-EVs in Idiopathic Premature Ovarian Failure | Iran | The patient is anesthetized and placed in a lithotomy position, after preparing and washing the vagina with normal saline, under transvaginal ultrasound guidance (Aloka-40000 vaginal probe, Japan) using needle puncture (Reproline medical Gmbh, Rheinbach/Germany). The injection of 2 ml of extracellular vesicles derived from mscs (equivalent to 30 million cells) will be performed into one ovary of the patient (the accessible ovary). | N | <https://clinicaltrials.gov/study/NCT06202547> |
| NCT05116761 | ExoFlo™ Infusion for Post-Acute COVID-19 and Chronic Post-COVID-19 Syndrome | N/A | Experimental:AC Treatment group  Normal saline 85 ml and exoflo 15 ml, which is 10.5 x 10^8^ EV | N | <https://clinicaltrials.gov/study/NCT05116761> |
| NCT04902183 | Safety and Efficacy of Exosomes Overexpressing CD24 in Two Doses for Patients With Moderate or Severe COVID-19 | Greece | Evaluate the safety and efficacy of exosomes overexpressing CD24 of two doses, Dose 1 - 10^9^ exosome Particles (per dose) versus Dose 2 - 10^10^ exosome Particles (per dose). The exosomes will be diluted in normal saline for inhalation via mouthpiece nebulization, administered once daily (QD) for 5 days. | N | <https://clinicaltrials.gov/study/NCT04902183> |
| NCT04602442 | Safety and Efficiency of Method of Exosome Inhalation in COVID-19 Associated Pneumonia | Russian Federation | Twice a day during 10 days inhalation of 3 ml special solution contained 0.5-2x10^10^ of nanoParticles (exosomes) of the first type and the second type. | N | <https://clinicaltrials.gov/study/NCT04602442> |
| NCT04747574 | Evaluation of the Safety of CD24-Exosomes in Patients With COVID-19 Infection | Israel | Group 1, 5 patients are treated with 1x10^8^ exosome Particles per 2 ml saline.  Group 2: 5 patients are treated with 5x10^8^ exosome Particles per 2 ml saline.  Group 3: 20 patients are treated with 1x10^9^ exosomes Particles per 2 ml saline.  Group 4: 5 patients are treated with 1x10^10^ exosomes Particles per 2 ml saline.  The drμg is aerosolized in normal saline for inhalation and administered via a standard hospital-grade inhalation device, QD for 5 days. Study treatment is given as an add-on to the standard of care. | N | <https://clinicaltrials.gov/study/NCT04747574> |
| NCT04491240 | Evaluation of Safety and Efficiency of Method of Exosome Inhalation in SARS-CoV-2 Associated Pneumonia. | Russian Federation | Twice a day during 10 days inhalation of 3 ml special solution contained 0.5-2x10^10^ of nanoParticles (exosomes) of the first type and the second type. | Y | <https://clinicaltrials.gov/study/NCT04491240> |
| NCT04366063/IRCT20200217046526N2 | Mesenchymal Stem Cell Therapy for SARS-CoV-2-related Acute Respiratory Distress Syndrome | Iran | The patients allocated randomly to three groups:  Control (n=20). Patients will conventional therapy for virus treatment and supportive care for ARDS will be used as control.  Intrvention Group1 (n=20). Patients will receive two doses of mscs 100×10e6 (±10%), at Day 0 and Day 2 intravenously.  Intervention Group 2 (n=20). Patients will receive two doses of mscs 100×10e6 (±10%), at Day 0 and Day 2 plus two doses of extracellular vesicles (evs) on Day 4 and Day 6 intravenously. | N | <https://clinicaltrials.gov/study/NCT04366063> |
| ISRCTN33578935 | To study the treatment of COVID-19 with severe viral pneumonia by using purified stem cell exosomes | Germany | Intravenous infusion of purified exosomes, xoglo®, which are isolated, neonatal, mesenchymal stem cell-derived extracellular vesicles at a dose of 0.2 mg/kg each in a total of 15ml on day 1 and day 3.  Control:15ml of saline, i.v. On Day 1 and Day 3 | N | <https://www.isrctn.com/ISRCTN33578935> |
| IRCT20190101042197N2 | valuation of the Safety and Efficiency of Mesenchymal Stem Cell Derived Exosomes in patients with ARDS of COVID-19; An interventional randomized double-blind controlled clinical trial: phase I and II | Iran | Intervention 1: Intervention group: Phase one: 12 patients with COVID-19 in two groups of 6 with Acute Respiratory Distress Syndrome (ARDS, each group includes intervention groups of 3). Phase Two: 60 patients with COVID-19-ARDS in two groups of 30 controls and intervention. Classified A and B in both control and intervention groups - Exosome receiving group (intervention groups) • Injection material: Umbilical Cord Stem Cell-derived exosomes• Injection rate: 1 Billion exosomes per kilogram of body weight • Injection carrier: Saline • Injection site: Intravenous. Intervention 2: Control group: Conventional treatments used in Acute Respiratory Distress Syndrome. | N | <https://irct.behdasht.gov.ir/trial/55380> |
| IRCT20200217046526N2 | Mesenchymal Stem Cell Therapy for Acute Respiratory Distress Syndrome in Coronavirus Infection: A Phase 2-3 Clinical Trial | Iran | The patients allocated randomly to tree groups: 1) Intervention 1, Patients will receive two doses of mscs. 3) Intervention 2, Patients will receive two doses of mscs intravenously plus two doses of evs. 3) Control, Patients will receive conventional therapy.the two intervention groups received two consecutive injections of mscs (100 × 10^6^ cells) or one dose of mscs (100 × 10^6^ cells) followed by one dose of MSC-derived extracellular vesicles (evs) | Y | <https://stemcellres.biomedcentral.com/articles/10.1186/s13287-023-03402-8?utm_source=xmol&utm_medium=affiliate&utm_content=meta&utm_campaign=DDCN_1_GL01_metadata> |
| IRCT20201202049568N3 | Evaluation of the Safety and Efficiency of human Umbilical Cord Derived Mesenchymal Stem Cell Exosomes in patients with ARDS of COVID-19; An interventional randomized double-blind controlled clinical trial: phase I and II | Iran | 1- Intravenous injection of 2ml normal saline contains umbilical cord mesenchymal stem cell-derived Exosomes 2- Intravenous injection of 2ml normal saline | N | <https://irct.behdasht.gov.ir/trial/54758> |
